# A case report of mucinous adenocarcinoma derived from intra-ampullary papillary-tubular neoplasm with a malignant course

**DOI:** 10.1186/s40792-020-01045-y

**Published:** 2021-01-15

**Authors:** Hiroaki Fujita, Keinosuke Ishido, Norihisa Kimura, Taiichi Wakiya, Hayato Nagase, Tadashi Yoshizawa, Toshihiro Haga, Shintaro Goto, Hiroshi Kijima, Kenichi Hakamada

**Affiliations:** 1 Department of Gastroenterological Surgery, Hirosaki University Graduate School of Medicine, Hirosaki, Japan; 2 Department of Pathology and Bioscience, Hirosaki University Graduate School of Medicine, Hirosaki, Japan

**Keywords:** Intra-ampullary papillary-tubular neoplasm, IAPN, Mucinous adenocarcinoma, Vater papilla, Liver metastasis

## Abstract

**Background:**

Intra-ampullary papillary-tubular neoplasm (IAPN) has been classified as a Vater papillary tumor. The prognosis of IAPN is generally relatively good. Here, we describe a patient with a mucinous adenocarcinoma cluster in the Vater papilla of IAPN origin.

**Clinical presentation:**

The patient was a 66-year-old man who was admitted to our hospital after a diagnosis of pancreatic head carcinoma based on a pancreatic duct dilatation found on abdominal ultrasound. CT showed a 40 mm lesion in the pancreatic head and expansion of the main pancreatic duct to a maximum diameter of 9 mm on the caudal side of the lesion. The extrahepatic bile duct had also expanded to a maximum diameter of 8 mm. PET/CT showed fluorodeoxyglucose (FDG) accumulation of SUV_max_ 6.02 that corresponded to the tumor in the pancreatic head, though it did not suggest distant metastasis. The patient was diagnosed with pancreatic head carcinoma T3 N0 M0 Stage IIA and underwent a pancreaticoduodenectomy. Pathology indicated that the tumor in the pancreatic head was a benign inflammatory lesion. On the other hand, the papillotubular tumor pervading the lumen in the duodenal papillary common channel met the criteria for IAPN, and a mucinous adenocarcinoma cluster found in the surrounding stroma suggested malignant transformation of IAPN. No metastasis to lymph nodes was demonstrated. With regard to the mucus phenotype of each lesion, the IAPN was MUC2 and MUC5AC positive, while the mucinous adenocarcinoma was MUC2-positive and MUC5AC-negative. In addition, CD10 was negative in both lesions, suggesting that mucus transformation from the gastric type to the intestinal type was a key element. A blood test 10 months after surgery showed increased CA19-9 (105 U/mL) and CEA (7.1 ng/mL). Abdominal CT showed multiple cystoid nodes in the liver, which were diagnosed as multiple liver metastases of mucinous adenocarcinoma transformed from the IAPN.

**Conclusions:**

We reported a case with IAPN that developed in the Vater papilla, which took an extremely malignant course. IAPN generally has a good prognosis, but it is important to understand that a malignant course may occur.

## Background

Vater papillary tumors vary grossly and histologically due to the anatomical complexity of papilla of Vater. The tumors are pathologically classified into three groups: (1) tumors similar to colon cancer, (2) tumors similar to cholangiocarcinoma and pancreatic ductal carcinoma, and (3) intraductal papillary mucinous neoplasm (IPMN), intraductal papillary neoplasm of the bile duct (IPNB), and intracholecystic papillary-tubular neoplasm (ICPN).

Ohike et al. proposed that intra-ampullary papillary-tubular neoplasm (IAPN) should also be included in group (3) in the above classification of Vater papillary tumors. IAPN is a precancerous lesion that may result in invasive cancer, but its prognosis is better than the standard ampullary carcinoma [1]. Here, we report a case of mucinous adenocarcinoma in the Vater papilla in which the origin of the tumor was IAPN; and we include a literature review. This is a rare case in which IAPN underwent a malignant transformation to a mucinous adenocarcinoma cluster with subsequent multiple liver metastases.

## Clinical presentation

The patient was a 66-year-old man who was diagnosed with pancreatic head carcinoma due to pancreatic duct dilatation on abdominal ultrasound and was admitted to our hospital. He had a history of chronic kidney disease (CKD) caused by IgA nephropathy and had received regular hemodialysis. He had no relevant family history. Blood tests on admission showed serum amylase 1273 U/L, γ-GTP 792 IU/L, AST 84 IU/L, ALT 283 IU/L, and ALP 1675 IU/L. With regard to renal function, serum blood urea nitrogen (BUN) and creatine levels were 42 mg/dL and 7.39 mg/dL, respectively. As for tumor markers, serum CA19-9, CEA, Span-1, and DUPAN-2 were 54 ng/dL, 3.1 ng/mL, 20.4U/mL, and 31.0U/mL, respectively.

**Fig. 1:**
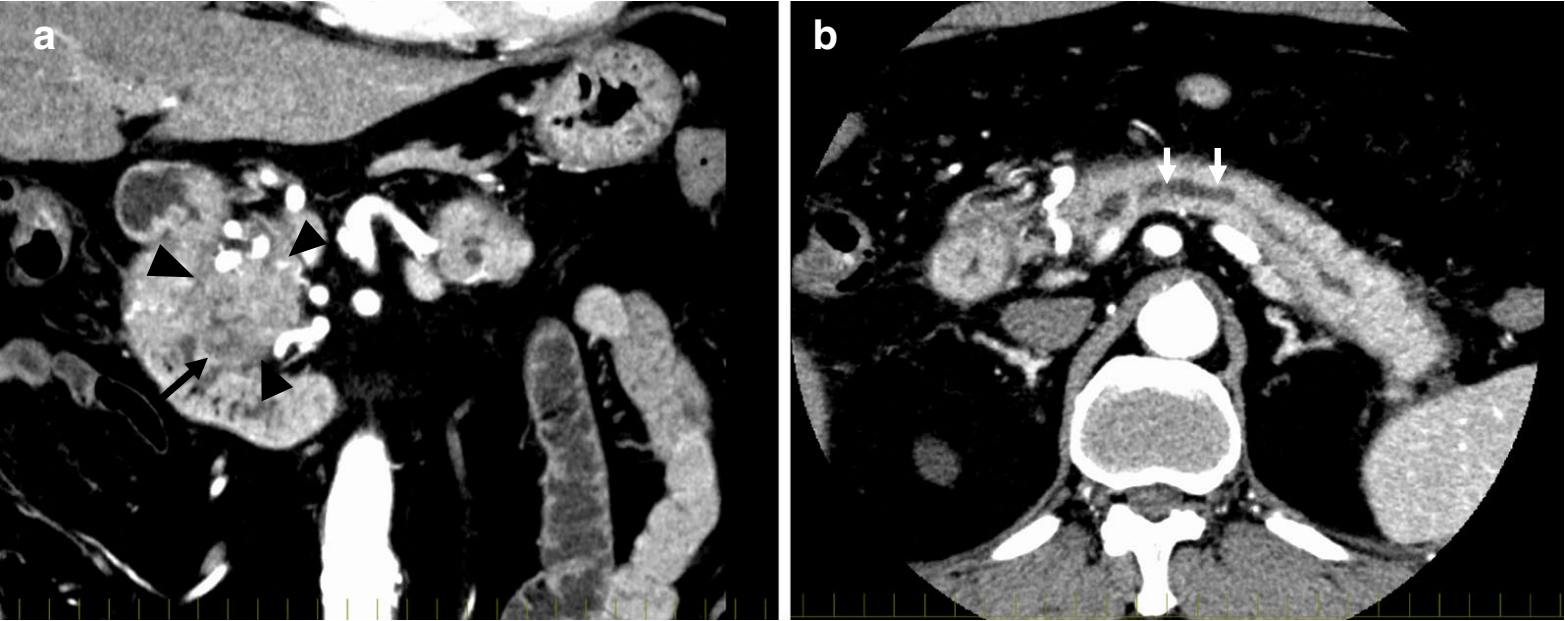
Contrast-enhanced CT. **a** A 40-mm mass with an irregular margin was detected in the pancreatic head (arrowhead), and a low-density area with 20 mm in diameter was observed on the duodenal side of the mass (arrow). **b** An expanding main pancreatic duct was detected with a 9-mm diameter at the distal side of the tumor (arrow)

**Fig. 2:**
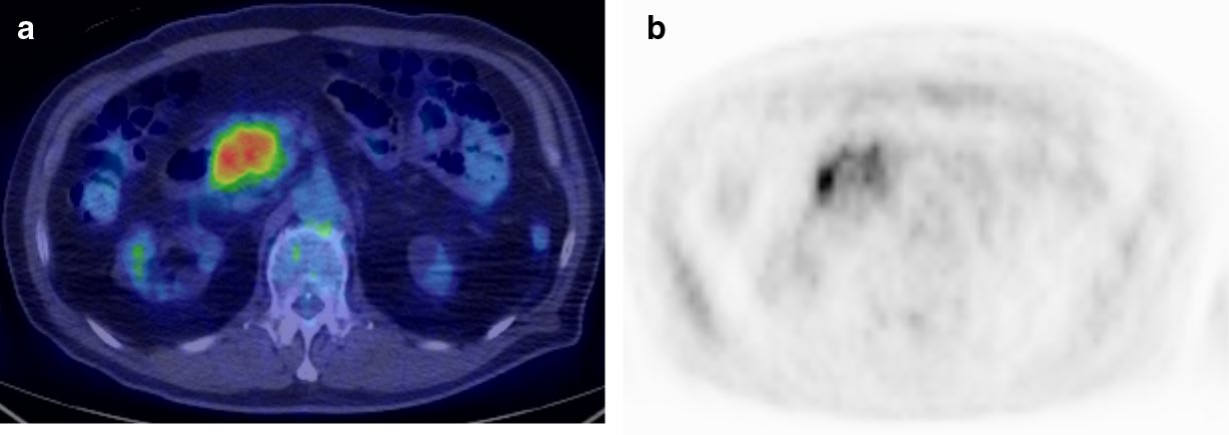
PET/CT. **a** PET/CT showed positive accumulation of SUVmax 6.02 at the lesion site; **b** the highest accumulation of about SUV 6.0 was observed near the duodenal side of the mass, and the lowest one at about SUV 3.0 was observed on the opposite side

CT showed a 40 mm mass with an irregular margin in the pancreatic head. It also showed that the main pancreatic duct of the distal pancreas was dilated to 9 mm in diameter. The density of the mass was not uniform, and a low-density area with 20 mm in diameter was observed on the duodenal side of the mass. The dynamic study showed that the pancreatic head mass had a slow contrast pattern, which was considered to be typical for pancreatic cancer (Fig. 1). Upper gastrointestinal endoscopy showed no abnormal findings such as lesions or mucous excretion in the Vater papilla. PET/CT showed positive accumulation of SUVmax6.02 on the pancreatic head mass. However, the distribution of FDG accumulation was not uniform. The highest accumulation of about SUV 6.0 was observed near the duodenal side of the mass, and the lowest one at about SUV 3.0 was observed on the opposite side (Fig. 2). Tumor biopsy using endoscopic ultrasonography (EUS) was not performed, because the patient did not agree to undergo this procedure. The patient was diagnosed with pancreatic head carcinoma T3 N0 M0 Stage IIA and underwent a pancreaticoduodenectomy. The intraoperative frozen-section diagnosis showed no tumor cell infiltration at the resection stump of the pancreas. However, a diagnosis of the bile duct stump was not performed. Inflammatory changes around the pancreas were prominent due to a pancreatitis. The operation time was 546 min and the blood loss was 1800 mL.

**Fig. 3:**
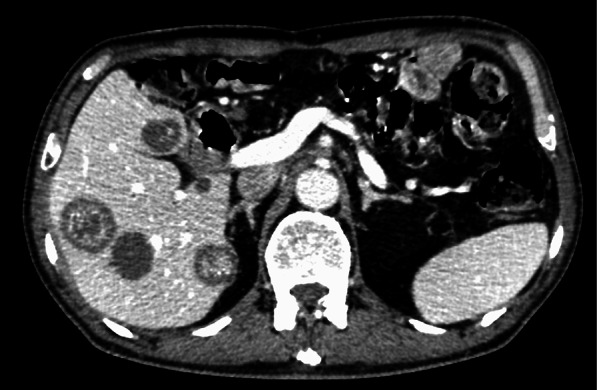
Contrast-enhanced CT. Multiple low-density areas in the liver were revealed. Cystoid nodes and irregular tumors with internal high density were found

There were no severe postoperative complications exceeding Grade 2 of the Clavien–Dindo classification, and the patient was discharged on postoperative day 23. He was then followed up without postoperative adjuvant chemotherapy due to CKD. A blood test 10 months after surgery showed increased CA19-9 (105 U/mL) and CEA (7.1 ng/mL). CT showed multiple low-density areas in the liver (Fig. 3). Cystoid nodes and irregular tumors with internal high density were found. These CT images led to the diagnosis of multiple liver metastases derived from the mucinous adenocarcinoma in the Vater papilla. Tumor biopsy was not performed due to the high risk of dissemination, and chemotherapy with gemcitabine was then planned. Although the chemotherapy was continued for 7 months, it failed because of the disease progression. His overall general condition would not allow the patient to continue the treatment. As a result, the patient has chosen palliative care.

### Pathological findings

**Fig. 4:**
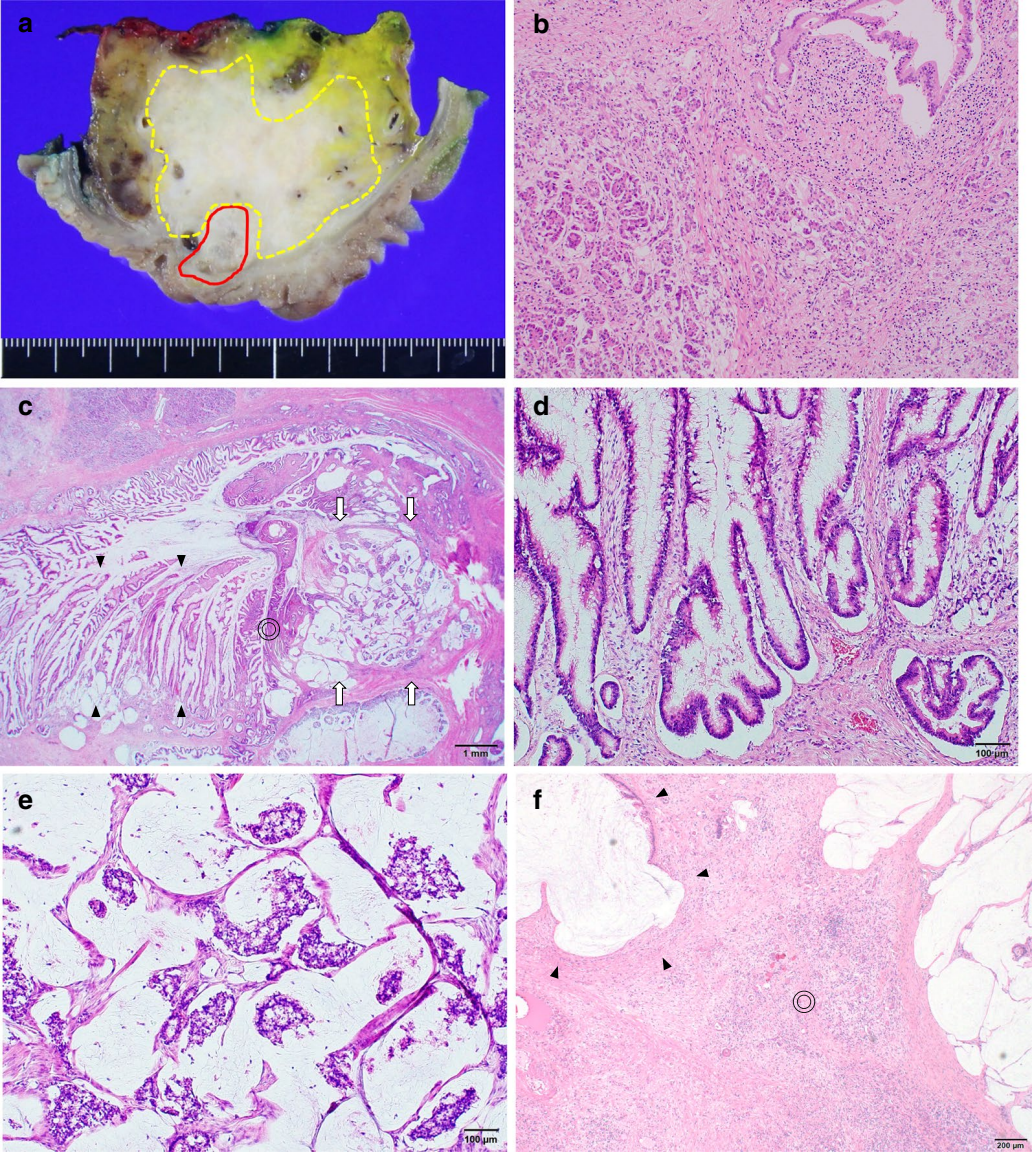
Pathology specimen. **a** Gross cross-section of the pancreatic head, showing a tumor lesion (solid line) pervading a white lesion with obscure margins (dotted line) and the lumen of the duodenal papillary common channel. **b** Mid-magnification image (HE staining ×40) of the white lesion (dots in **a**). Atrophic pancreatic tissues with fibrogenesis and inflammatory cell infiltration. **c** Low-magnification image (HE staining ×12.5) of the duodenal papillary common channel (solid line in **a**). In the sphincter of Oddi, a papillotubular tumor pervading the lumen was observed (arrowhead). A mucinous nodular lesion (arrow) was found in the stroma, with transiting images of the tumor (double circle). **d** High-magnification image (HE staining ×100) of the arrowhead in **c**. Hyperplasia of tumor cells with findings of papillotubular growth, IAPN, and low-to-high-grade dysplastic adenocarcinoma. **e** High-magnification image (HE staining ×100) of the arrow in **c**. The lesion formed multiple neoplastic mucinous nodes, indicating findings of mucinous carcinoma derived from IAPN. **f** Mucinous adenocarcinoma infiltrating to the parenchyma of the fibrotic pancreas (HE staining ×40). Mucinous adenocarcinoma (arrowhead) was infiltrating to the parenchyma of the pancreas (double circle). The pancreatic parenchyma was excessively atrophic with the inflammatory cell infiltration

**Fig. 5:**
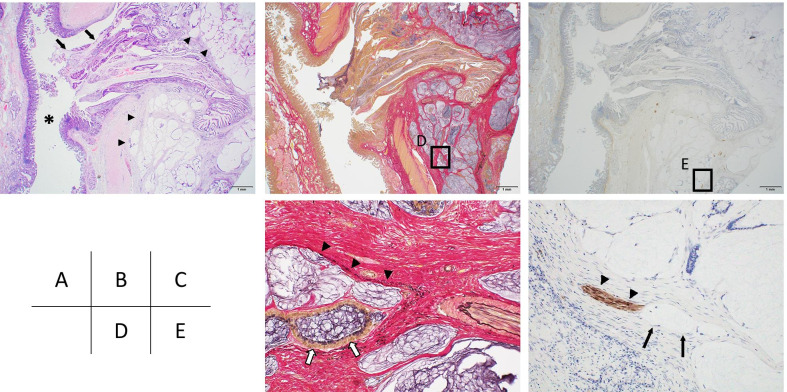
Elastica van Gieson staining and S-100 immunostaining. The duodenal papillary common channel was investigated with HE staining (**a** ×12.5), Elastica van Gieson staining (**b** ×12.5; **d** ×100), and S-100 immunostaining (**c** ×12.5; **e** ×100). **a** The IAPN (arrow) pervading in the common channel (asterisk) was transformed to the mucinous adenocarcinoma which invaded to the stroma of Vater papilla extensively (arrowhead). **d** Elastica van Gieson staining (high-magnification image of square in **b**) showed cancer cells (arrow) inside elastic tissues in the vein wall (arrowhead), indicating venous invasion. **e** S-100 immunostaining (high-magnification image of square in **c**) showed mucous nodes (arrow), adjacent to a nerve fiber bundle (arrowhead), indicating perineural infiltration

**Fig. 6:**
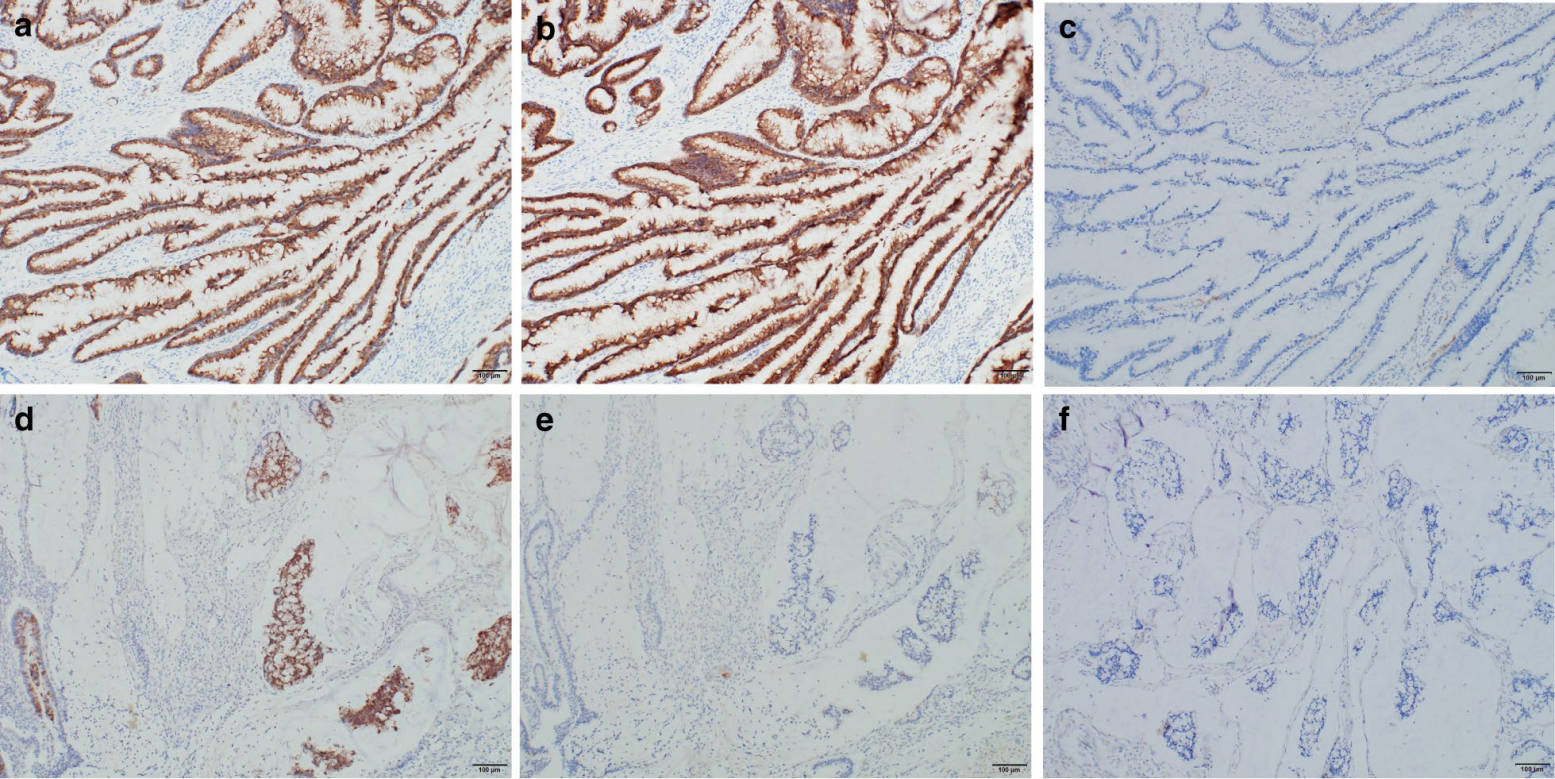
Immunostaining for the mucinous phenotype. Immunostaining showed positive findings for **a** MUC2, **b** MUC5AC, and negative finding for **c** CD10 in the papillotubular tumor site. On the other hand, **d** positive MUC2, **e** negative MUC5AC, and **f** negative CD10 were revealed at the mucinous adenocarcinoma site

The tumor in the pancreatic head was a white, 55 × 40 mm lesion with grossly obscure margins (Fig. 4a). Pathological findings suggested that the white lesion comprised atrophic pancreatic tissues with fibrogenesis and inflammatory cell infiltration, but with no neoplastic changes; consequently, the lesion was considered to be an inflammatory tumor (Fig. 4b). IgG4 immunostaining confirmed the non-inflammatory tumor, indicating the absence of autoimmune pancreatitis. A 20 × 11 mm neoplastic lesion had pervaded the lumen along the duodenal papillary common channel (Fig. 4a). Furthermore, it was comprised of papillary-tubular tumor and mucinous nodular lesions infiltrating the sphincter of Oddi (Fig. 4c). The papillary tumor had eosinophilic reticula in subcircular nuclei with crude chromatin and a papillary and tubular structure (Fig. 4d). Tumor cells showed various morphologies, including low-grade dysplasia and high-grade dysplasia with high cellular atypia. These futures were consistent with IAPN as reported by Ohike et al. Most of the lesion was IAPN developing in the common channel, but some parts formed multiple neoplastic mucinous nodes in the sphincter of Oddi, which indicated invasive mucinous adenocarcinoma (Fig. 4e). Furthermore, the mucinous adenocarcinoma had partially invaded to the parenchyma of the pancreas (Fig. 4f). We evaluated the specimen with Elastica van Gieson staining and S-100 immunostaining to elucidate venous and perineural invasions (Fig. 5a–c). As a result, a venous invasion was found in the mucinous adenocarcinoma cluster, and nerves were also partially found in the mucous nodes, which suggested perineural infiltration into the tumor cells (Fig. 5d, e). The mucoid phenotype of the IAPN was positive for MUC2 and MUC5AC (Fig. 6a, b). In contrast, invasive mucinous carcinoma was positive for MUC2 and negative for MUC5AC (Fig. 6d, e). Both lesions were negative for CD10 (Fig. 6c, f). Based on all of these results, the patient was diagnosed with IAPN associated with malignant transformation to mucinous adenocarcinoma. The stage as ampullary carcinoma was determined to be T3 N0 M0 Stage IIA based on the UICC classification ver. 7.

## Discussion

The concept of IAPN was proposed by Ohike et al. in 2010 [1, 2], with the disease defined as being similar to intraductal papillary neoplasms [3–6] occurring in the Vater papilla. IAPN develops mainly in the duodenal papillary common channel, and then forms a papillary, polypoidal, or pervading glandular neoplasm. Histological findings indicate mixed atypical cells, including hyperplasia, adenoma, and high-grade dysplasia, and therefore, IAPN is regarded as precancerous ampullary carcinoma [7]. The diagnostic criteria for IAPN are: (1) preinvasive (dysplastic), (2) papillary or polypoidal mass-forming development, (3) distinguishable from adjacent luminal mucosa, and (4) limited to inside the papilla, and limited to the common channel or adjacent distal biliary and pancreatic ducts. The prognosis of non-invasive IAPN is quite good, and 5-year survival rate was reported to be 100%. Even invasive IAPN still has a better outcome compared to typical ampullary adenocarcinoma (mean survival: 51 vs. 31 months, P < 0.01) [1]. Our case had a neoplastic papillotubular tumor centered on the common channel, and most of the lesion was intraepithelial neoplasia in the lumen of the common channel. These findings match the diagnostic criteria for IAPN. Mucinous adenocarcinoma components were present in the stroma, and these were flanked by IAPN. These findings suggest that the mucinous adenocarcinoma cluster was derived from IAPN.

The comorbid lesion in the pancreatic head was diagnosed as an inflammatory tumor pathologically... Ohike et al. suggested that IAPN causes obstruction of the common channel due to distending growth, showing marked expansion in the upper biliary and pancreatic duct [1, 2]. With IAPN, abdominal pain, nausea, jaundice, and diabetes are more likely to occur in comparison with other intraductal papillary tract neoplasms [8]. Therefore, it is highly likely that the pancreatic duct was obstructed by IAPN, and tumor-forming pancreatitis occurred in our case. CT showed a 40 mm mass with an irregular margin in the pancreatic head, which contained a low-density area (LDA) that was 20 mm in diameter on the duodenal side. According to the pathological results, the 20 mm LDA could have been an indication of an IAPN. A strong accumulation, SUVmax 6.02, was observed in the pancreatic head mass, and diagnosed as pancreatic cancer preoperatively. However, pathologically, it was confirmed to be an inflammatory mass. Although FDG accumulation is often observed on PET-CT in mass-forming pancreatitis; therefore, SUV 2.1–3.0 is usually thought of as the cut-off value when differentiating between this and pancreatic cancer [9]. In our case, since a very high accumulation of SUV_max_ 6.02 was observed in the pancreatic head mass, clinicians would generally rule out pancreatitis. The higher SUV value, however, was observed near the duodenal side of the mass, and the comparatively low SUV value was observed on the opposite side. Therefore, it is highly likely that the high SUV value reflected IAPN and the mucinous adenocarcinoma around the Vater papilla, and the low SUV reflected the mass-forming pancreatitis in the pancreatic head.

Ohike et al. defined IAPN as a precancerous lesion and found that the prognosis of invasive IAPN is markedly better than that of typical papillary carcinoma [1]. IAPN is reported to be the counterpart of IPMN. Intraductal papillary mucinous carcinoma (IPMC) has a better prognosis than pancreatic ductal adenocarcinoma (PDAC) [10]. The better prognosis of IPMC is due to the following nature: (1) IPMC does not have an inherent aggressive biology with higher incidence of invasion and metastasis unlike PDAC. (2) IPMC is more likely to be visualized in image examinations earlier than PDAC. As a result, early detection and therapeutic intervention are possible. Due to its similar pathophysiology, it is considered that IAPN has a better prognosis than other types of carcinoma in the Vater papilla. However, our case had an extremely malignant course. For extrahepatic cholangiocarcinoma, Carriaga et al. found 5-year survival rates of mucinous carcinoma which was 2.9% with the poorest prognosis among all tissue types [11]. Mucinous adenocarcinoma in our case was associated with highly aggressive characteristics that caused early multiple liver metastases. To elucidate the relationship of such a high-grade mucinous adenocarcinoma cluster and IAPN, the mucoid phenotype was examined. The component of IAPN was a gastrointestinal mixed type that had a positive mucoid phenotype for MUC2 and MUC5AC, and a negative one for CD10. On the other hand, mucinous adenocarcinoma component was positive for MUC2, and negative for MUC5AC and CD10, indicating the intestinal type [12, 13]. The immunohistological findings suggested that the mucoid phenotype of the IAPN had mutated from a gastrointestinal mixed type to an intestinal type. Colonic carcinomas with MUC2-positive and MUC5AC-negative were increased the risk of the nodal metastasis, leading to a poor prognosis [14, 15]. The intestinal type with CD10 positive was more likely to develop venous invasion and liver metastasis [16–18]. However, in our case, CD10 was negative for mucinous adenocarcinoma component. Therefore, a highly aggressive course in our case was assumed to be caused by the malignant potential unrelated to CD10. There have been no reports associating invasive IAPN with a poor prognostic course. Therefore, this report is considered to be quite rare.

## Conclusions

We reported a case with IAPN that developed in the Vater papilla, which took an extremely malignant course. IAPN generally has a good prognosis, but it is important to understand that a malignant course may occur.

## Data Availability

Data will be made available by the corresponding author upon reasonable request.

## Abbreviations

IAPN: Intra-ampullary papillary-tubular neoplasm
IPMN: Intraductal papillary mucinous neoplasm
IPNB: Intraductal papillary neoplasm of the bile duct
ICPN: Intracholecystic papillary-tubular neoplasm
CKD: Chronic kidney disease
EUS: Endoscopic ultrasonography
IPMC: Intraductal papillary mucinous carcinoma
PDAC: Pancreatic ductal adenocarcinoma
